# Inhibition of microRNA‐222 up‐regulates TIMP3 to promotes osteogenic differentiation of MSCs from fracture rats with type 2 diabetes mellitus

**DOI:** 10.1111/jcmm.14777

**Published:** 2019-11-06

**Authors:** Chenyi Jiang, Wenyang Xia, Tianyi Wu, Chenhao Pan, Haojie Shan, Feng Wang, Zubin Zhou, Xiaowei Yu

**Affiliations:** ^1^ Department of Orthopaedic Surgery Shanghai Jiao Tong University Affiliated Sixth People’s Hospital Shanghai China

**Keywords:** fracture healing, mesenchymal stem cells differentiation, miR‐222, streptozotocin, type 2 diabetes mellitus

## Abstract

Type 2 diabetes mellitus (T2DM) is the most common diabetes and has numerous complications. Recent studies demonstrated that T2DM compromises bone fracture healing in which miR‐222 might be involved. Furthermore, tissue inhibitor of metalloproteinase 3 (TIMP‐3) that is the target of miR‐222 accelerates fracture healing. Therefore, we assume that miR‐222 could inhibit TIMP‐3 expression. Eight‐week‐old rats were operated femoral fracture or sham, following the injection of streptozotocin (STZ) to induce diabetes one week later in fractured rats, and then, new generated tissues were collected for measuring the expression of miR‐222 and TIMP‐3. Rat mesenchymal stem cells (MSCs) were isolated and treated with miR‐222 mimic or inhibitor to analyse osteogenic differentiation. MiR‐222 was increased in fractured rats and further induced in diabetic rats. In contrast, TIMP‐3 was reduced in fractured and further down‐regulated in diabetic rats. Luciferase report assay indicated miR‐222 directly binds and mediated TIMP‐3. Furthermore, osteogenic differentiation was suppressed by miR‐222 mimic and promoted by miR‐222 inhibitor. miR‐222 is a key regulator that is promoted in STZ‐induced diabetic rats, and it binds to TIMP3 to reduce TIMP‐3 expression and suppressed MSCs’ differentiation.

## 1 INTRODUCTION

Diabetes mellitus (DM), commonly known as diabetes, is a metabolic disorder disease that is characterized by high blood glucose levels in the body over a prolonged period. Diabetes is caused by either not enough insulin, which is a hormone produced by pancreas that regulates blood glucose metabolized for energy, or insulin‐resistant cells which cannot respond properly to insulin. The former is type 1 diabetes (T1DM), an autoimmune disease that can be effectively treated by supplementary insulin, and the latter is type 2 diabetes mellitus (T2DM). There are also other types of diabetes, such as gestational diabetes and monogenic diabetes.

According to the World Health Organization (WHO), T2DM is the most common diabetes that accounts for 90% of all the cases (more than 422 million) who are suffering from DM. DM is the seventh leading cause of death in the world since 2016. T2DM can shorten 10‐year expectance partly due to its numerous complications,^1^ including two to four times risk of cardiovascular disease, like ischaemic heart disease and stroke, a 20‐fold increase in lower limb amputations and elevated rates of hospitalizations. In the developed countries and increasing elsewhere, T2DM is the largest cause of non‐traumatic blindness and kidney failure.^2^, ^3^ It also has a negative impact on cognitive dysfunction and dementia like Alzheimer's disease and vascular dementia.^4^ Nowadays, more and more evidence suggested that diabetes could compromise the skeletal system, causing detrimental bone effects like bone quality deterioration, loss of bone strength, elevated fracture risk and impaired bone fracture healing.^4^, ^5^, ^6^, ^7^, ^8^, ^9^ However, the mechanism how diabetes adversely affects bone fracture healing is still not fully elucidated; thus, there is no effective treatment for accelerating bone healing in diabetic patients.

MicroRNA‐222 (miR‐222) is one of the microRNAs that are non‐coding RNAs and involved in post‐transcriptional regulation of gene expression by altering both the stability and translation of mRNAs. Recently, several studies demonstrated that miR‐222 participated in the process of vascular formation,^10^, ^11^ which is a crucial part for bone healing. As followed, miR‐222 was discovered to inhibit bone fracture healing^8^ and significantly up‐regulated in diabetic rats compared with wild‐type rats.^12^ These all suggest that miR‐222 was involved in the processing of fracture healing impaired by diabetes.

Tissue inhibitor of metalloproteinase 3 (TIMP‐3), also known as metalloproteinase inhibitor 3, is one of the tissue inhibitors of matrix metalloproteinases that degrade extracellular matrix (ECM). TIMP‐3 facilitates in bone formation,^13^ bone fracture healing, and overexpressed TIMP‐3 could contribute to recovery from diabetes.^14^ Moreover, TIMP‐3 might be the target of miR‐222.^15^, ^16^ Therefore, we assume that miR‐222 negatively affects bone fracture healing by mediating TIMP‐3 in diabetes, and we used femoral fracture rat model injected with STZ to induce diabetes, then treated with miR‐222 mimic or inhibitor to support our hypothesis.

## 2 METHOD AND MATERIALS

### 2.1 Animal model

All experiments were approved by the Institutional Animal Care and Use Committee in Shanghai Jiao Tong University Affiliated Sixth People's Hospital. Surgery was performed as described before.^17^, ^18^ Briefly, after intramuscular injection of penicillin (50 IU/kg), an incision lateral to the right knee was made to expose the articular surface of the distal femur in 8‐week‐old female Sprague Dawley rats under anaesthesia. A 10‐mm gauge needle was inserted in the medullary canal for fixation, and the tip and the distal end were flattered for rotation stability, and then, the incision was closed followed a blunt trauma to generate the mid‐diaphyseal fracture with a falling 500 g weight from 30 cm height over a three‐point bending mechanism. After one week, fractured rats were randomly divided into two groups. One was intraperitoneally injected with a low dose of streptozotocin (STZ, Sigma Chemical Company) dissolved in 0.1 mol/L sodium citrate buffer (pH 4.4) for one week to induce diabetes, and the other group was injected with buffer for control.^9^ At the different time‐points, rats were killed, and femurs were collected with the removal of the muscle and soft connective tissue.

Blood was collected from rat tails, and blood glucose and insulin levels were measured using commercially available kits according to the manufacturer's instructions, respectively (BioScience).

### 2.2 Luciferase report assay

A fragment of the 3’‐UTR of TIMP‐3 mRNA or mutated one containing the putative miR‐222 binding sequences was cloned into the firefly luciferase reporter construct pmiR‐RB‐Repost (Ribobio). Human embryonic Kidney 293T cells (HEK293T cells) were used for reporter assay by seeding into 96‐well plates and cotransfected with TIMP3‐3’UTR‐Luc (1 µg) or mutated TIMP3‐3’UTR and miR‐222 or miR‐negative control (NC) by lipofectamine 2000. After 48‐hour incubation, luciferase activity was analysed on a scintillation counter by a dual‐luciferase reporter system (GeneCopoeia, Luc‐Pair Duo‐Luciferase Assay kit).^16^

### 2.3 Isolation and culture of rat bone marrow mesenchymal stem cells (MSCs)

The rats were killed after injecting STZ for 6 weeks into fractured rats, and then, bilateral bones at the fracture site were collected. DMEM (Dulbecco's modified Eagle medium, Thermo Fisher Scientific) was aspirated into the medullary cavity repeatedly with a syringe, so that the bone marrow cells were washed out and dispersed sufficiently to form a single cell suspension. Then, MSCs were cultured in the DMEM containing 10% foetal bovine serum (FBS) in an incubator with 5% CO_2_ at 37°C. After primary culture for 24 or 48 hours, the medium was changed to remove the unattached cells. When the cells were confluent up to 80% to 90%, 0.25% trypsin (Thermo Fisher Scientific) was used to passage the cells into 6‐well plates at a ratio of 1:2.^19^ For phenotypic characterization, the P4 MSCs were trypsinized, centrifuged and then incubated with anti‐CD34, anti‐CD45, anti‐CD29 and anti‐CD44 antibodies in PBS with 1% foetal calf serum for 30 minutes at 4°C followed by flow cytometry. MSCs were starved for 8 hours, and then, 50 nmol/L miR‐222 mimic (BIONEER, Almeda, CA, USA), 100 nmol/L miR‐222 inhibitor (Qiagen) or a non‐functional negative control (NC) was transfected into MSCs with lipofectamine RNAimax.^8^

### 2.4 TIMP‐3 siRNA knockdown in MSCs

After MSCs were collected 24 hours, TIMP‐3 Stealth Select RNAi or Stealth RNAi Negative Control (Invitrogen) with miR‐222 inhibitor (Qiagen) was transfected into MSCs using the Lipofectamine RNAimax reagent (Invitrogen). After transfected 48 hours, cells were harvested for Western blot and RT‐PCR.

### 2.5 ALP vitality testing

After seven days of MSC’s incubation, cells were centrifuged and assayed for ALP activity according to the kit instruction. Briefly, medium was removed, and ALP was stained using a mixture of 0.1 g/L Naphthol AS‐MX alkaline phosphatase solution and 0.6 g/L Fast Blue RR Salt. After 10 minutes in the dark, the vitality assay was read.^19^

### 2.6 Real‐time PCR

Briefly, total RNA was extracted from either new generated tissues of rats or MSCs with miRNeasy Mini Kit (Qiagen). For the evaluation of miR‐222 level, mature miRNA was reverse transcribed with Bulge‐Loop mRNA qPCR Primer (Ribobio) prior to qPCR according to the manufacturer's instructions, and qRT‐PCR for miR‐222 cDNA synthesis was performed with iScript cDNA Synthesis kit (Bio‐Rad). The sequences of primers are as follows: MiR‐222 forward 5′‐AGCTACATCTGGCTACTGGGT‐3′ and reverse 5′‐GCGAGCACAGAATTAATACGAC‐3′; TIMP3 forward 5′‐GCUAUCAGUCCAAACACUATT and reverse UAGUGUUUGGACUGAUAGCTT; GADPH forward: 5′‐ATCACCATCTTCCAGGAGGGA‐3; and reverse 5′‐ CCTTCTCCATGGTGGTGAAGAC‐3.^15^ ALP forward 5’‐ACACCTTGACTGTGGTTTACTGCTGA‐3′ and reverse 5′‐CCTTGTAGCCAGGCCCGTTA‐3′; Runx2 forward 5’‐TTCTCCAACCCACGCCTGCAC‐3′ and reverse 5′‐CAGGTACGTGGTAGTGAGT‐3′.

### 2.7 Western blot

At the end‐point of experiment, MSCs were collected, and then, protein was extracted by RIPA buffer. The concentration of protein was measured by bicinchoninic acid (BCA) (Beyotime); 40 mg of each protein sample was added in SDS‐PAGE gel and then transferred to PVDF membrane (Thermo Fisher Scientific). The membrane was blocked, incubated with primary antibody, then secondary antibody. Finally, image exposure was performed to observe the protein expression. All the antibodies were purchased from Abcam.

### 2.8 Statistical analysis

Data were analysed by statistical product and service solutions (SPSS 16.0, SPSS Inc) and expressed as means ± SD. Non‐parametric test was used to compare the difference between two groups, and one‐way analysis of variance followed by Least Significant Difference as its post hoc test was used to compare multiple groups.

## 3 RESULTS

### 3.1 The expression of miR‐222 and TIMP‐3 was altered during fracture healing in T2DM rats

**Figure 1:**
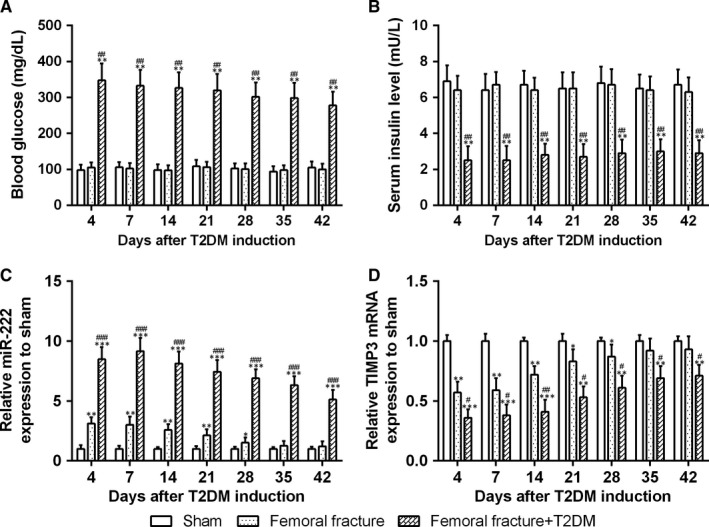
Blood glucose (A) and serum insulin level (B) in fracture rats after T2DM induction and relative expression levels of miR‐222 and TIMP3 in newly generated tissue at the fracture site. qRT‐PCR was used to measure the miR‐222 (C) and TIMP3 (D) expressions during the bone healing after T2DM induction. The relative expression patterns were compared to GAPDH, then normalized to sham group. n = 8 for each group. Data are presented as means ± SD *P < .05, **P < .01 and ***P < .001 compared to sham group, #*P* < .05, ##*P* < .01 and ###*P* < .001 compared to femoral fracture group

First, 8‐week‐old rats were operated femoral fracture or sham as reported before. After one week, fractured rats were randomly divided into two groups. One group was injected with STZ to induce T2DM, and the other with vehicle. Therefore, there are three groups in total, sham, femoral fracture and femoral fracture + T2DM. We monitored the blood glucose level, serum insulin, miR222 expression and TIMP‐3 expression, respectively, after the induction of diabetes from day 4 to day 42 during the bone healing. Blood glucose levels were significantly elevated after T2DM induction compared with the other two groups and remained high level for the following days (Figure 1A). In the opposite, insulin levels were remarkably reduced in T2DM group and remained low compared with the other groups (Figure 1B). These suggested the success of T2DM rat model.

To measure the expression of miR‐222 during the bone healing, we collected the newly generated tissues at the fracture site at the different time‐points after STZ induction. On the fourth day of STZ treated, miR‐222 expression in femoral fracture group was significantly increased compared with sham group (Figure 1C), and then, the expression of miR‐222 kept falling down till back to normal on the Day 35 as a result of bone healing. All these data indicated that miR‐222 was involved in bone fracture and bone healing. T2DM rats displayed a dramatic increase in miR‐222 expression on Day 4, which demonstrated that diabetes could further up‐regulate the expression of miR‐222 in the femoral fracture rats. Although miR‐222 was time‐dependently decreased in the development of bone healing, on Day 35 when the non‐diabetic rats were already recovered from bone fracture, the expression of miR‐222 remained high in the T2DM rats, which demonstrated that T2DM rats had a longer bone‐healing period that is consistent with previous paper that diabetes could inhibit bone healing.

In contrast, compared with sham, the expression of TIMP‐3 was decreased after femoral fracture and time‐dependently increased, then went back to normal on Day 35 the same day when the expression of miR‐222 was reversed (Figure 1D). Moreover, T2DM could further diminish the expression of TIMP‐3 and slightly reverse in a time‐dependent way, but at the end time‐point Day 42, the expression of TIMP‐3 was still remarkably lower than that in vehicle‐treated femoral fracture rats. Taken together, miR‐222 and TIMP‐3 are all related to bone healing in the opposite way.

**Figure 2:**
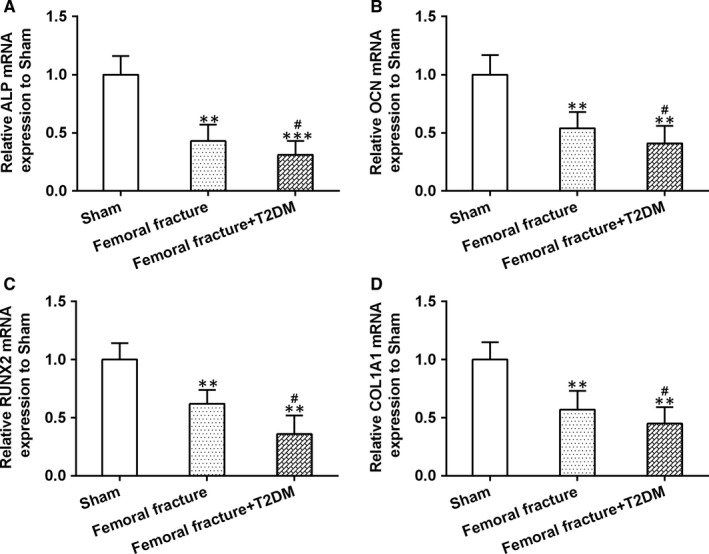
Fracture rats after T2DM induction showed delayed fracture healing. The mRNA expression of osteoblast‐related genes, ALP (A), OCN (B), RUNX2 (C) and COLIA1 (D) in newly generated tissue at the fracture site, were measured by qRT‐PCR. n = 8 for each group. Data are presented as means ± SD. **P < .01 and ***P < .001 compared to sham group, #*P* < .05 compared to femoral fracture group

To better clarify the delayed bone healing in T2DM rats, we measured the expression of osteoblast‐related genes including alkaline phosphatase (ALP), osteocalcin (OCN), runt‐related transcription factor 2 (RUNX2) and collagen a1 (COLIA1) in newly generated tissue at the fracture site. All these four genes were decreased in femoral fracture group and further down‐regulated after T2DM induction (Figure 2A‐D). These data demonstrated that T2DM impaired bone fracture healing.

### 3.2 TIMP‐3 is a direct target of miR‐222

**Figure 3:**
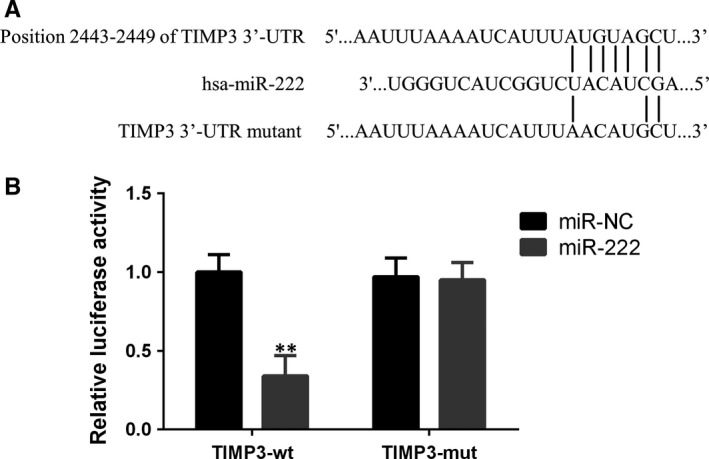
TIMP3 is a direct target of miR‐222. A, The suspected binding of mature human miR‐222 with the wild‐type 3′‐UTR region of TIMP3 mRNA is shown. A mutated 3′‐UTR of TIMP3 is also shown. B, A dual‐luciferase reporter assay was performed with human embryonic kidney 293T cells (HEK293T cells) cotransfected with firefly luciferase constructs containing wild‐type 3′‐UTR region of TIMP3 (TIMP3‐wt) or mutated 3′‐UTR region of TTMP3 (TIMP3‐mut) and miR‐222 mimic or control. The relative luciferase activities were evaluated 24 h after transfection. Data are presented as means ± SD. **P < .01 compared to miR‐NC

Previous publications showed that TIMP‐3 might be the target of miR‐222. To testify whether TIMP‐3 directly binds to miR‐222, we analysed the mRNA sequence of TIMP‐3 and found out the potential binding position (2443‐2449), which might directly bind to miR‐222. To further prove that hypothesis, we constructed two plasmids: one contains wild‐type 3’‐UTR region of TIMP‐3 (TIMP3‐wt), and the other contains mutated 3’‐UTR region (TIMP3‐mut). The latter was mutated on sites 2444‐2447 as shown in Figure 3A. Luciferase reporter assay discovered that miR222 mimic could significantly decrease the luciferase activity of TIMP‐3 compared with miR‐negative control, while this inhibitory effect caused by miR‐222 was totally blocked in the mutated TIMP‐3 (Figure 3B). Taken together, TIMP‐3 is a target of miR‐222 by directly binding to miR‐222 on position 2443‐ 2449.

### 3.3 miR‐222 reduces the expression of TIMP‐3 in rat MSCs

**Figure 4:**
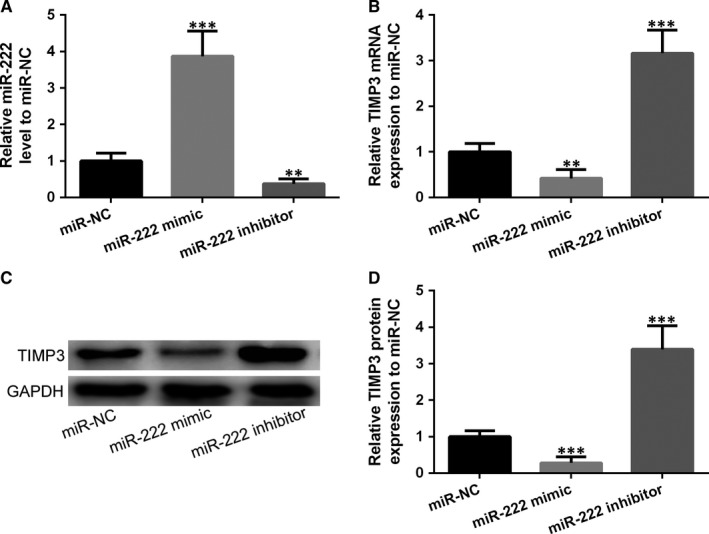
MSCs were infected by miR‐222 mimic, miR‐222 inhibitor or miR‐NC. The mRNA expression of miR‐222 (A), mRNA expression of TIMP‐3 (B) and protein expression of TIMP‐3 (C and D) after overexpression miR‐222 (miR‐222 mimic) and knockdown miR‐222 (miR‐222 inhibitor). Data are presented as means ± SD. **P < .01 and ***P < .001 compared to miR‐NC group

We isolated MSCs from STZ‐induced diabetic rats that suffered bone fractured for 6 weeks described in detailed material and method, and the phenotype of MSCs was measured by FACS (Figure S1). MSCs were treated with miR‐222 mimic or its inhibitor. Figure 4A showed the effectiveness of either overexpressing miR‐222 by adding miR‐222 mimic or knocking down by miR‐222 inhibitor. As expected, both mRNA and protein expressions of TIMP‐3 were reduced by miR‐222 mimic and induced by miR‐222 inhibitor (Figure 4B‐D). Altogether, miR‐222 directly binds to TIMP‐3 to reduce both mRNA and protein expression of TIMP‐3 in MSCs, and on the contrast, suppressing miR‐222 could enhance TIMP‐3 expression.

### 3.4 Inhibition of miR‐222 promoted osteogenic differentiation of MSCs

**Figure 5:**
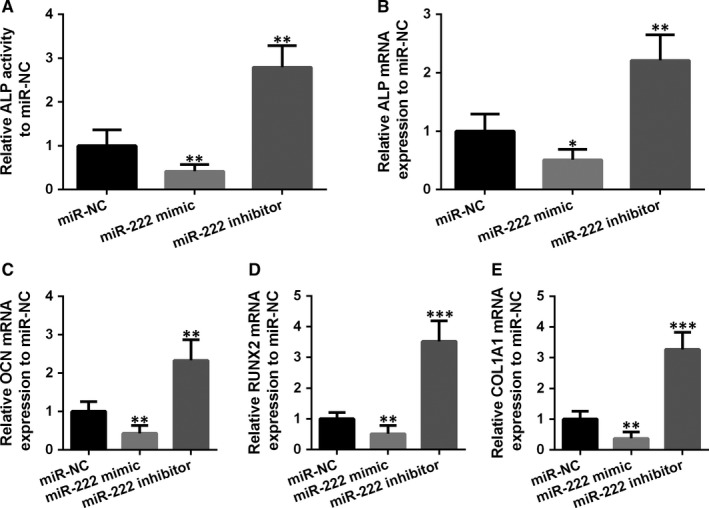
Inhibition of miR‐222 promoted osteogenic differentiation of MSCs. A, The cell ALP activity was measured after inhibition of miR‐222, or miR‐222 overexpression. B‐E, The mRNA expression of osteoblast‐related genes, ALP, OCN, RUNX2 and COLIA1 treated with miR‐222 mimic or miR‐222 inhibitor. Data are presented as means ± SD. *P < .05, **P < .01 and ***P < .001 compared to miR‐NC group

**Figure 6:**
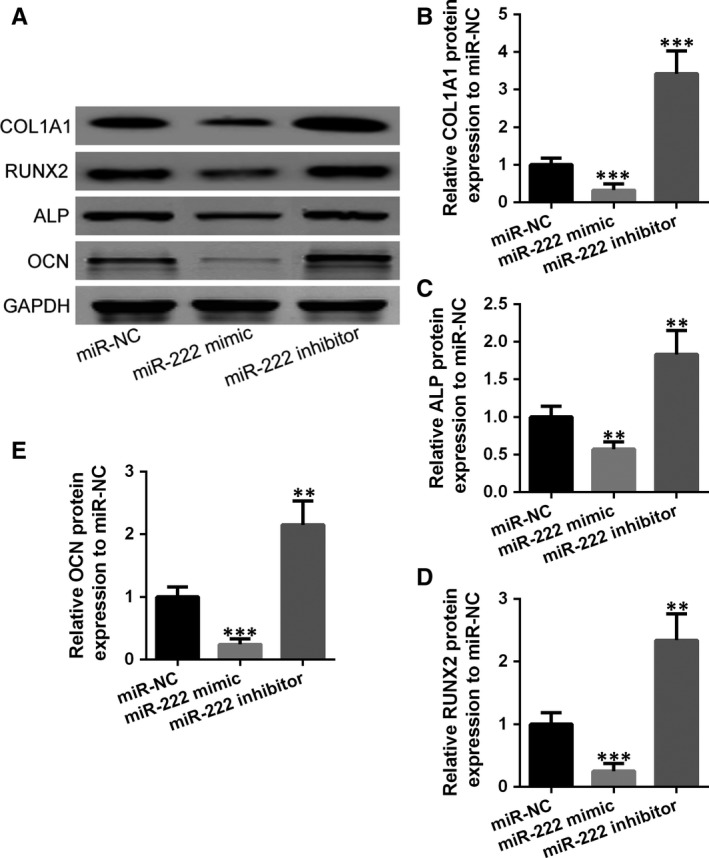
Inhibition of miR‐222 promoted osteoblast‐associated proteins expression in MSCs. A, Western blotting was used to measure the protein expressions of COL1A1, RUNX2, ALP and OCN, and the relative expression was normalized to miR‐NC group (B‐E). Data are presented as means ± SD. **P < .01 and ***P < .001 compared to miR‐NC group

Alkaline phosphatase (ALP) is a biomarker of fracture healing progression and used for predicting fracture healing, and thus, we measured the ALP activity in the MSCs. Unsurprisingly, after overexpression of miR‐222, ALP activity in MSCs was reduced, as well as the expression of osteogenesis‐related genes, including OCN, RUNX2 and OLIA1 (Figure 5A‐E). On the contrary, opposite results were observed with miR‐222 inhibitor that all these oestrogenic genes were enhanced. In addition, overexpression of miR‐222 brought down the expression of osteoblast‐associated proteins such as ALP, Runx2, OCN and COLIA1, while inhibiting miR‐222 significantly enhanced the expression of above proteins (Figure 6A‐D). All the above results suggested that miR‐222 suppressed osteogenic differentiation of MSCs.

### 3.5 TIMP3 inhibited miR‐222 to regulate osteogenic differentiation of MSCs

**Figure 7:**
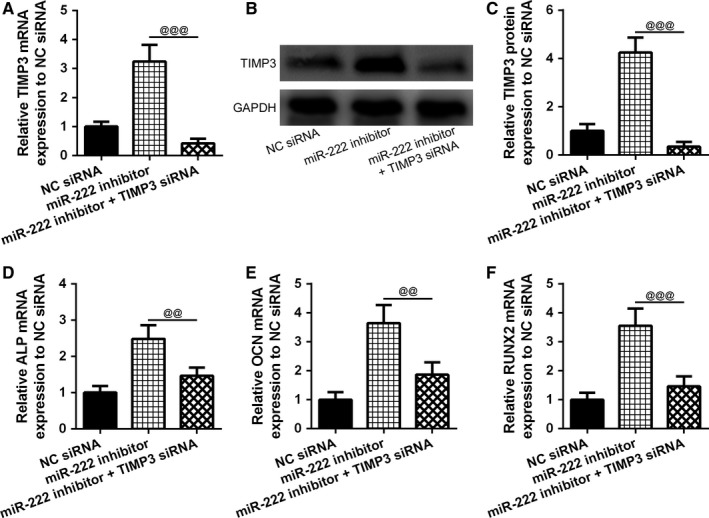
TIMP3 mediated the effects of inhibition miR‐222 on the regulation of osteogenic differentiation of MSCs. A, Inhibition of miR‐222 promoted the mRNA expression of TIMP3, which was reversed by TIMP3 siRNA. (B and C) Inhibition of miR‐222 promoted the protein expression of TIMP3, which was reversed by TIMP3 siRNA. D‐F, Inhibition of miR‐222 promoted the mRNA expressions of osteoblast‐related genes ALP, OCN and RUNX2, which was reversed by TIMP3 siRNA. Data are presented as mean ± SD. **P < .01 and ***P < .001 compared to miR‐NC group

In MSCs, both the mRNA and protein expressions of TIMPs were significantly revoked by miR0222 inhibitor, which was reversed by TIMP‐3 siRNA (Figure 7A‐C). Furthermore, to support our hypothesis that inhibition of TIMP‐3 could suppress osteogenic differentiation, we also measured the expression of osteogenic related genes. As expected, mRNA expression of ALP, OCN and RUNX2, which was induced by miR‐222 inhibitor, was remarkably reduced by TIMP‐2 siRNA (Figure 7D‐F). All these data suggested that TIMP‐3 was suppressed by miR‐222, which led to the inhibition of MSCs’ differentiation. In summary, miR‐222 could regulate osteogenic differentiation by directly binding and inhibiting TIMP‐3 in T2DM rats.

## 4 DISCUSSION

Type 2 diabetes is a complicated metabolic disorder disease that has numerous complications. Gradual studies demonstrated that diabetes could delay bone fracture healing in which diabetic patients suffer longer recovery time from bone regeneration. Bone fracture healing, as well as bone formation, involves in the process of the secretion of osteoblasts matrix, fibre surrounding, formation of osteoid and deposition of calcium. Numerous studies demonstrated several mechanisms that how diabetes significantly impaired bone healing and tried to provide new strategies to treat delayed bone healing in diabetic patients.^5^ For example, progranulin (PGRN), a growth factor‐like molecule that induced chondrogenesis, improved fracture healing in T1DM mice.^11^ Forkhead box protein O1 (FOXO‐1) in chondrocytes that blocked the premature removal of cartilage associated with bone formation rescued the diabetic effect on bone healing.^20^, ^21^ Moreover, transplanting bone marrow stromal cells transfected with CKCL13 could facilitate bone healing in diabetic rats.^22^ Parathyroid hormone (1‐34) promotes fracture healing in ovariectomized rats with T2DM.^9^ Except all kinds of potential drugs for treating delayed bone healing in diabetes, Dr Tsuchiya's group discovered that methylglyoxal (MGO), which is reactive intermediate derivatives of glucose metabolism, caused impaired osteoblastic differentiation to delay bone repair in diabetes.^23^ Recently, some publications recognize the role of miRNAs in the regulation of bone fracture healing by rapidly and efficiently binding to the target sites to degrade target genes or reduce the translation of target proteins so as to mediate osteoblast generation. MiR‐210 was reported to promote the differentiation of MSCs into osteoblasts in mouse.^24^ Moreover, miR‐347b also facilitated fracture healing.

In addition, a few publications documented the use of miRNA inhibitor or mimic for bone formation in vivo. Bone marrow MSCs transfected with miR‐26a mimics enable the entire repair of the critical size calvarial bone defect via improving angiogenesis and osteogenesis.^25^ Besides systemic and local administration of miR‐92a promoted fracture healing by angiogenesis in the mouse femoral fracture model.^26^ Moreover, adipose tissue‐derived stem cells knockdown miR‐31.^27^ Combined with beta‐tricalcium phosphate scaffolds were capable of repairing a rat severe‐sized calvarial defect. Our study demonstrated that miR‐222 was enhanced after fracture, and further increased in the diabetic rats. On Day 35 when fracture was healing and miR‐222 went back to normal level in wide‐type rats, miR‐222 still dramatically promoted in diabetic rats. T2DM further enhanced miR‐222 expression that might be the reason why diabetes delayed fracture healing due to the dramatic elevated expression of miR‐222.

Bone marrow–derived MSCs are multipotential stromal cells, which is a kind of stem cell that can self‐renew and differentiate in multi‐direction under different conditions. MSCs can switch into variety of cell types, including osteoblasts (bone cells), chondrocytes (cartilage cells), myocytes (muscle cells), adipocytes that give rise to marrow adipose tissue,^7^, ^28^ ectodermal neurons and endodermal hepatocytes.^29^ The mechanism of MSCs differentiation is still not clear yet, but it is well known that this differentiation can be regulated by many factors, including transcriptional factors, environment factors, chemical factors and interaction between cells. Considering that miR‐222 inhibitor could improve MSCs differentiation into osteoblasts, these all provide us a theoretical basis and point out a research direction for future therapeutic options of fracture healing.

TIMPs consist of TIMP‐1, TIMP‐2, TIMP‐3 and TIMP‐4.^13^ TIMP‐3, as all the other TIMPs, is a strong inhibitor of angiogenesis.^14^ It is located on the membrane and can bind with extracellular matrix for adhesion onto basal membrane,^30^ thus reducing matrix degradation caused by matrix metallopeptidase 9 (MMP‐9) increase.^31^ Furthermore, MMP‐9, regulated by TIMP‐3 and produced by osteoclasts, has been identified as a stimulator for angiogenesis in the bone microenvironment.^32^ A deficiency in TIMP‐3 might directly block inhibition of angiogenesis and further generate an excess of MMP‐9, which in turn results in increased bone angiogenesis and vascularization. In particular, TIMP‐3 binds directly to vascular endothelial growth factor (VEGF) receptor 2, blocking the action of VEGF on endothelial cells.^33^ Lack of TIMP‐3 is detrimental to bone development and maintenance. Bone mechanics, architecture and composition in the skeleton were all altered in the TIMP‐3‐deficient mice.^34^ Taken together, TIMP‐3 plays an important role in bone formation, bone angiogenesis and bone fracture healing. In our study, we confirmed that TIMP‐3 is the target of miR‐222, and its expression was in the opposite way of miR‐222, increased by miR‐222 inhibitor and reduced by miR‐222 mimic.

However, there are still lots of questions that need to be further explored. Although we verified that TIMP‐3 is the target of miR‐222, TIMP‐3 was reported to promote fracture healing. It is still unknown that whether miR‐222 could affect bone fracture healing through binding and inhibiting TIMP‐3 as well as MSC differentiation in T2DM rats. This might need further more studies to investigate that. Furthermore, how TIMP‐3 facilitates fracture healing is not clear, either by suppressing matrix degradation via MMP‐9 reducing, or blocking VEGF, or affect another pathway that involved in the bone fracture healing, which needs further study. In our study, miR‐222 could inhibit TIMP‐3 expression and suppress MSCs differentiation into osteoblasts, but whether TIMP‐3 involved in the inhibition of MSC differentiation by miR‐222 is not investigated, and this can be further studied.

In our study, we used the rat model as published before. Rats were operated femoral fracture first, then injected with STZ to induce diabetes. This model aimed to simulate the type 2 diabetic patients who suffered bone fracture. However, it will be a better option that injecting STZ into rats to induce T2DM first, following femoral fracture. Otherwise, mouse model could be another option. C57BL mice are fed on high‐fat diet (60% fat) for two or three months to induce diabetes followed femoral fracture operation.

In summary, we found that miR‐222 and TIMP‐3 were altered in the opposite way in the processing of bone fracture healing; both of these can be further deepening in the diabetic rats. In fractured rats, miR‐222 and TIMP‐3 returned to normal level after bone healing; however, in the diabetic rats, both miR‐222 and TIMP‐3 still significantly changed when bone should be healed in the normal condition. Next, we confirmed TIMP‐3 is the target of miR‐222 and that miR‐222 could directly bind to TIMP‐3 and inhibit it. Furthermore, MSC differentiation is blocked by miR‐222 mimic and enhanced by miR‐222 inhibitor.

## CONFLICT OF INTEREST

The authors declared that they had no conflict of interest.

## AUTHOR CONTRIBUTION

Xiaowei Yu and Zubin Zhou involved in the data collection and manuscript writing; Chenyi Jiang, Wenyang Xia, Tianyi Wu, Chenhao Pan, Haojie Shan and Feng Wang involved in the data collection and analysis.

## Data Availability

Data could be obtained upon request to the corresponding author.
