# Supplementary material for: Inhibition of microRNA‐222 up‐regulates TIMP3 to promotes osteogenic differentiation of MSCs from fracture rats with type 2 diabetes mellitus
Source: J Cell Mol Med. 2019 Nov 6;24(1):686–94. doi: 10.1111/jcmm.14777 (PMC6933364; doi:10.1111/jcmm.14777)
Supplement: Supplementary file 1 [file JCMM-24-686-s001.docx]

**Supplementary Materials**





Figure S1. Characterization of phenotype of MSCs. FACS analysis of surface markers on MSCs.
